# Health related quality of life and mental distress after PCI: restoring a state of equilibrium

**DOI:** 10.1186/1477-7525-11-144

**Published:** 2013-08-27

**Authors:** Johann Sipötz, Oliver Friedrich, Stefan Höfer, Werner Benzer, Thomas Chatsakos, Georg Gaul

**Affiliations:** 1 Karl Landsteiner Institute for Scientific Research in Clinical Cardiology, Vienna, Austria; 2 Department of Cardiology, Hanusch Krankenhaus, Vienna, Austria; 3 Department of Medical Psychology, Innsbruck Medical University, Innsbruck, Austria; 4 Department of Interventional Cardiology, Academic Hospital Feldkirch, Feldkirch, Austria

**Keywords:** Health related quality of life, Mental distress, Coronary artery disease, Percutaneou*s* coronary intervention, MacNew, Hospital anxiety and Depression scale

## Abstract

**Background:**

Patient self reported measures for Health Related Quality of Life (HRQOL) and mental distress are frequently used to evaluate outcome of therapeutic strategies in cardiac patients.

Our study aims to describe changes in HRQOL and mental distress after percutaneous coronary intervention (PCI) focusing on temporal pattern of change and interdependences between both outcome measures.

**Method:**

163 PCI patients recruited at 7 cardiovascular care units in Austria answered MacNew Health Related Quality of Life and Hospital Anxiety and Depression Scale (HADS) questionnaires during hospital stay after intervention and at 1, 6, 12 and 24 months.

**Results:**

Improvement of MacNew HRQOL was found up to 6 month after PCI. Mental distress declined during the first month of the follow-up period. MacNew HRQOL is negatively correlated to mental distress. The relationship could be well described by a linear regression with MacNew HRQOL as dependent and HADS Total score as independent variable. The explained variance (R^2^) of the regression equation increases drastically from 45% at the baseline to a level between 67% and 77% in the follow up.

**Conclusion:**

Our data suggest that the regression equation describing the relation between MacNew HRQOL and HADS-Total score six month after PCI defines a state of equilibrium: In absence of actual symptoms of coronary artery disease (CAD) both measures reflect the general health status and the general attitude underlying the self-assessment of health. At the baseline this equilibrium is imbalanced because the symptoms of CAD have a more pronounced impact on the disease specific MacNew HRQOL measure than on the non-disease specific HADS measure for mental distress. In order to use the MacNew questionnaire as a monitoring and/or prognostic tool it seems promising to refer to the state of equilibrium to define expectancy values for successful treatment.

## Introduction

The basic idea of patient-reported outcome research is to integrate the patient’s point of view in a systematic way into clinical practice. There is a growing awareness among health care providers that the evaluation of medical strategies should include the perception of individual patients [1]. Mortality and morbidity have traditionally been the focus of outcome studies of existing and new therapies. Patient-centred treatment outcome measures such as HRQL questionnaires are now recommended in relevant research studies as well as in clinical care by the European Medicines Agency [2] and the US Food and Drug Administration [3]. A recent consensus statement from the Society for Cardiovascular Angiography and Interventions called for the use of quality of life outcome in clinical trials, appropriate use criteria, practice guidelines, and reimbursement policies for percutaneous coronary intervention (PCI) [4].

In patients suffering from coronary artery disease (CAD) the MacNew patient-reported health related questionnaire is a well-established instrument for assessing Quality of Life [5]. Previous studies have shown, that MacNew Health Related Quality of Life (HRQOL) is eligible to describe the impact of CAD and the effect of CAD treatment strategies [6-9]. Pedersen and colleagues reported that MacNew HRQOL is predictive for early cardiac events after PCI [10].

An important finding of previous studies on MacNew HRQOL in CAD patients was that symptoms of mental distress (anxiety and depression) measured by the patient-reported Hospital Anxiety and Depression Scale (HADS) are not only closely related to MacNew HRQOL, but are even more important determinants of MacNew HRQOL scores than variables describing the severity of CAD [11]. Some studies have shown that improvement of MacNew HRQOL after treatment of CAD by PCI, coronary artery bypass surgery (CABG) or medical treatment is accompanied by a reduction of the level of mental distress [6,12,13]. To the best of our knowledge there is up to now no study investigating the relationship between HRQOL and mental distress in PCI patients in a longitudinal study design, although it seems highly likely that a medical intervention targeting CAD has a much more pronounced impact on disease specific HRQOL than on mental distress, which implies that the interrelation between these two health status measures must alter after PCI.

Our study aims to describe the change of MacNew and HADS scores and their interdependent relationship in a sample of PCI patients over a 24 months follow-up period in detail to gain a thorough understanding of the temporal pattern of change and the respective interrelation between Quality of Life and mental distress.

## Method

### Study sample

The study sample was drawn from the PRODES-Austria registry in which between January 1st, 2008 and December 31st, 2011 310 PCI patients form 7 centres in Austria aged between 18 and 80 years treated with a drug eluting Xience stent participated. Patients with acute ST-elevation myocardial infarction (STEMI) or stent deployment during the last 6 months were excluded from the registry. The PRODES-Austria registry was designed to analyse changes in Health related Quality of Life and Mental Distress after PCI over a 24 months follow-up period by a prospective, multi-centre approach. Clinical data were collected at hospital admission. The patients completed the baseline questionnaires (HADS and MacNew) during hospital stay after cardiovascular intervention. Follow-up questionnaires were answered at 1, 6, 12 and 24 months. The study was conducted in accordance to the Helsinki Declaration with approval by the local ethic commission. All participants provided written informed consent.

### MacNew

The self-administered MacNew is designed to assess patient’s perception about how CAD affects daily functioning and contains 27 items with a global HRQOL score and physical limitation, emotional, and social function subscales. Using a 2-week time frame, the MacNew items and subscales are scored from 1 (low HRQL) to 7 (high HRQL). To assess the impact of PCI on individual patients we refer to the minimal important difference (MID), the smallest change of a score patients perceive as important [14]. For the MacNew Global score Dixon and colleagues have established a MID of 0.5 points [15]. The German version of MacNew has been shown to be valid and reliable for ischemic heart disease [5].

### HADS

The Hospital Anxiety and Depression Scale (HADS) is a validated psychological screening instrument widely used in CAD patients [16,17]. The questionnaire includes 14 questions and results in separate scores for anxiety and depression. A recently published 10-year review questioned the ability of HADS to differentiate between anxiety and depression and recommended to use HADS scores more general as a measure for mental distress [18]. A score for mental distress can be calculated by summing up depression and anxiety scores [19].

### Statistical analysis

Discrete variables were compared with chi-square test or Fisher Exact test when appropriate. Continuous variables were examined with t-test or Mann–Whitney-U-Test, changes of MacNew and HADS scores with Wilcoxon test. Linear regressions were calculated using the ordinary least square (OLS) method. All statistical analyses were performed with PASW Statistics 18.0 software with two-tailed p-values <0.05 considered statistically significant.

## Results

### Patient characteristics

A total of 310 patients participated in the PRODES-Austria registry. For the present study we included all patients who completed all four follow-up questionnaires (N = 163). Table 1 summarizes the patient characteristics and shows a comparison between included and excluded patients. Patients excluded because of incomplete follow-up (N = 147) were less educated, more often obese and hypertensive but they did not differ significantly with regard to other available variables including baseline MacNew and HADS Total scores (Table 1).

**Table 1:** Patient characteristics

	**Included**	**Excluded**	**p**
	**N = 163**	**N = 147**	
Age	63.7 ± 9.2	63.1 ± 9.4	0.575
Gender (male)	73.0%	70.7%	0.695
Marital status			
single	12.9%	17.2%	0.212
married / life partnership	74.7%	65.5%	
widowed	12.3%	17.2	
missing	0.6%	1.4%	
Education			
≥12 years	27.2%	15.9%	0.019
missing	3.1%	6.1%	
Professional status			
employed	27.0%	27.1%	0.167
retired	66.9%	62.5%	
unemployed	1.8%	6.9%	
other	4.3	3.5%	
missing	-	2.0%	
Financial status			
satisfied with financial status	80.7%	78.5%	0.635
missing	1.2%	8.2%	
CCS classification			
0/1	17.3%	10.4%	0.256
2	48.3%	58.5%	
3	25.5%	23.7%	
4	9.0%	7.4%	
missing	11%	8.2%	
Single vessel intervention	84.7%	82.9%	0.670
missing	-	-	
AHA classification			
A	12.6%	4.7%	0.105
B1	30.5%	36.7	
B2	26.5%	30.5%	
C	30.5%	28.1%	
missing	7.4%	12.9%	
Hypercholesterolemia*	84.9%	86.8%	0.636
missing	2.5%	2.0%	
Hypertension*	60.2%	78.9%	<0.001
missing	1.2%	3.4%	
Diabetes mellitus*	21.7%	21.1%	0.911
missing	3.7%	3.4%	
Obesity (BMI ≥ 30)	23.3%	33.3%	0.039
missing	2.5%	4.1	
Smoking	29.6%	35.5%	0.275
missing	2.5%	4.1%	
MacNew Emotional	5.12 ± 1.11	5.02 ± 1.01	0.298
MacNew Physical	5.04 ± 1.16	5.00 ± 1.18	0.673
MacNew Social	5.33 ± 1.17	5.33 ± 1.12	0.809
MacNew Global	5.11 ± 1.06	5.04 ± 1.00	0.431
HADS Total	9.62 ± 6.79	10.42 ± 9.98	0.215

* treated

### Quality of life after PCI: change in MacNew HRQOL scores

**Figure 1:**
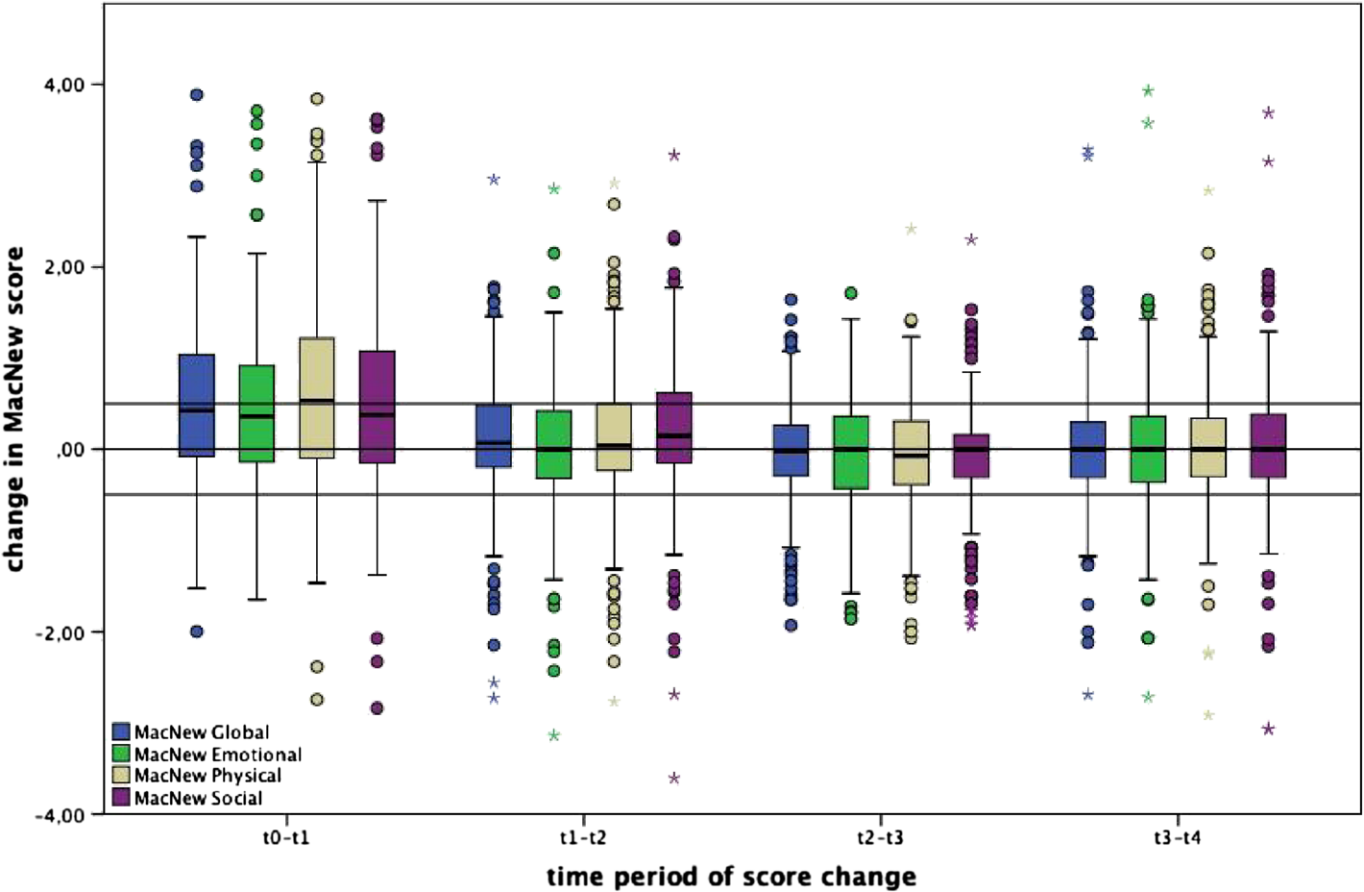
**Change in MacNew scores.** (t0: baseline; t1: 1 month; t2: 6 months; t3: 12 months, t4: 24 months). Boxes show median and quartiles, whiskers maximum within 1.5 interquartile range, dots outliers outside 1.5 interquartile range and stars outliers outside 3.0 interquartile range. Horizontal lines at 0.5 and −0.5 designate the range of clinical important change (≥0.5) in MacNew Global score.

Based on a significant improvement in all three MacNew subscales MacNew Global score increased significantly between baseline and 1-month follow-up (p < 0.001). Between 1 month and 6 months after PCI we found an additional significant improvement in MacNew Physical (p = 0.038) and MacNew Social (p < 0.001) but not in MacNew Emotional score. The resulting increase in MacNew Global score was just over the level of significance (p = 0.051). After 6 months up to the end of the follow-up period at 24 months MacNew scores remained stable (Table 2, Figure 1, for absolute score values see Additional file 1).

**Table 2:** Change of MacNew scores (t0: baseline; t1:1 month; t2: 6 months; t3: 12 months, t4: 24 months)

		**t0-t1**	**t1-t2**	**t2-t3**	**t3-t4**	p t0-t1*	p t1-t2*	p t2-t3*	p t3-t4*
MacNew Emotional	median	0.36	0	0	0	<0.001	0.480	0.861	0.837
	(Q1/Q3)	(−0.14/0.92)	(−0.35/0.43)	(−0.43/0.36)	(−0.36/0.36)				
	mean	0.47	0.02	−0.03	0.03				
	(SD)	(0.9)	(0.79)	(0.67)	(0.84)				
MacNew Physical	median	0.54	0.04	−0.07	0	<0.001	0.038	0.156	0.511
	(Q1/Q3)	(−0.12/1.23)	(−0.23/0.52)	(−0.39/0.31)	(−0.3/0.34)				
	mean	0.58	0.12	−0.09	0.04				
	(SD)	(1.11)	(0.86)	(0.69)	(0.75)				
MacNew Social	median	0.38	0.15	0	0	<0.001	<0.001	0.111	0.934
	(Q1/Q3)	(−0.15/1.08)	(−0.15/0.64)	(−0.33/0.17)	(−0.31/0.39)				
	mean	0.46	0.19	−0.1	0.02				
	(SD)	(1.09)	(0.87)	(0.67)	(0.86)				
MacNew Global	median	0.43	0.07	−0.02	0	<0.001	0.051	0.566	0.789
	(Q1/Q3)	(−0.08/1.04)	(−0.2/0.49)	(−0.29/0.27)	(−0.33/0.31)				
	mean	0.51	0.08	−0.06	0.04				
	(SD)	(0.91)	(0.76)	(0.62)	(0.75)				

* Wilcoxon-Test

**Figure 2:**
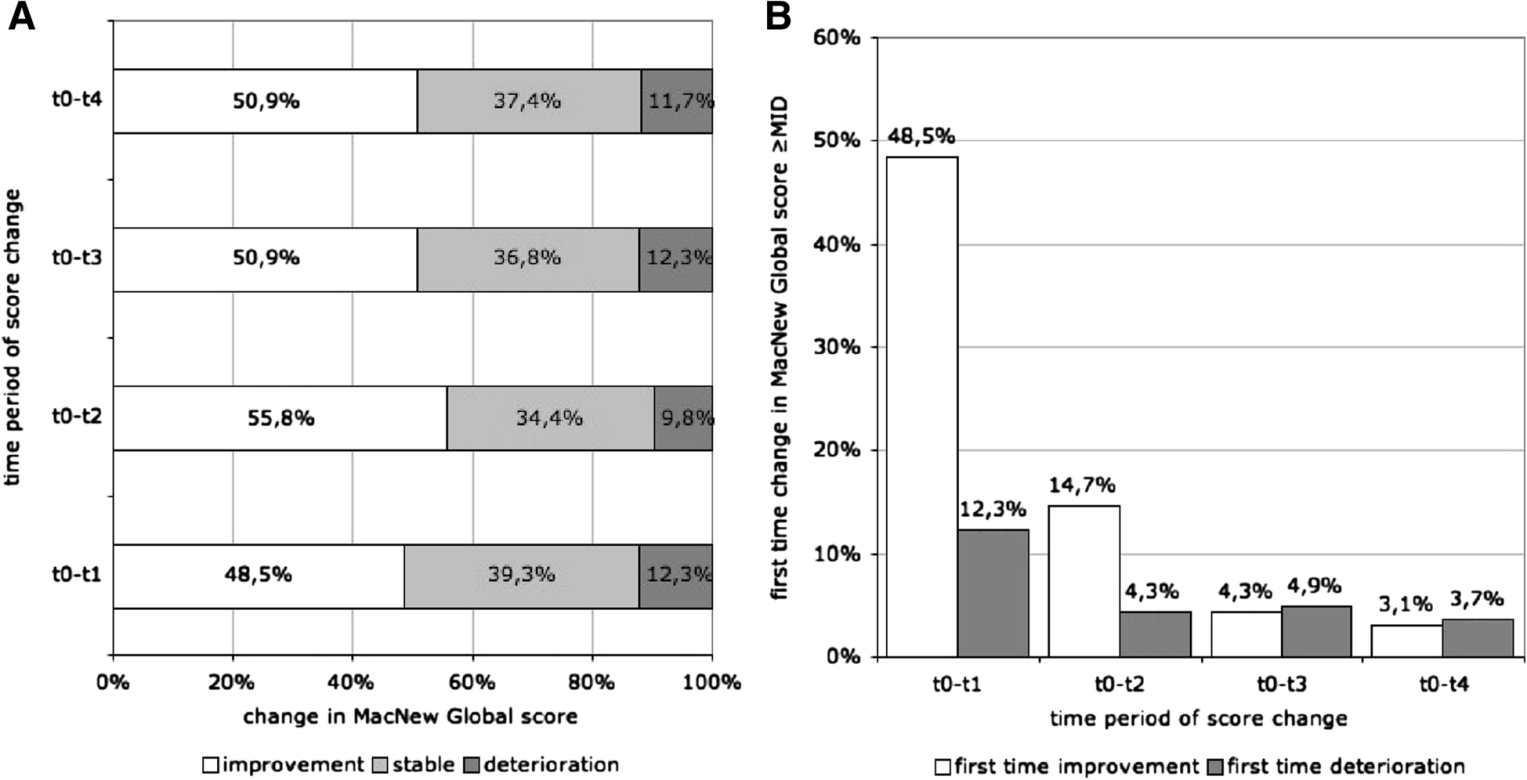
**Clinically important change in MacNew Global score (≥0.5) between baseline and follow-up.** Percentages of patients improving, deteriorating or staying stable **(A)** and percentages of patients improving or deteriorating for the first time relative to the baseline score at the respective point of time **(B)**. (t0: baseline; t1: 1 month; t2: 6 months; t3: 12 months, t4: 24 months).

The comparison of baseline MacNew Global scores to the four points of follow-up showed, that clinically relevant improvement of MacNew Global HRQOL (≥0.5) occurred in 48.5-55.8% of the patients. More than a third of the patients remained stable (34.4-39.3%). Clinically relevant deterioration (≥0.5) was observed in 9.8-12.3% (Figure 2A).

Looking at the temporal pattern of clinically relevant change in MacNew HRQOL we found that most of the patients improved immediately in the first month after PCI (48.5%). Another 14.7% experienced clinically relevant improvement up to the 6-months follow-up. At 12 and 24 months only 4.3% and 3.1%, respectively, showed clinically relevant first time improvement of MacNew HRQOL. Clinically relevant deterioration after PCI relative to the baseline was found in 12.3% up to 1-month follow-up. At 6, 12 and 24 months only 4.3%, 3.9%, and 3.7% clinically relevant deteriorating patients were registered (Figure 2B).

### Mental distress: change in HADS total score

With regard to mental distress measured by HADS-Total score we found a small but statistically significant decrease from baseline to 1-month follow-up (p = 0.020). Later on no significant change occurred (Table 3, for absolute score values see Additional file 1).

**Table 3:** Change of HADS total score (t0: baseline; t1:1 month; t2: 6 months; t3: 12 months, t4: 24 months)

	**t0-t1**	**t1-t2**	**t2-t3**	**t3-t4**	p t0-t1*	p t1-t2*	p t2-t3*	p t3-t4*
median	−1	0	0	0	0.020	0.272	0.342	0.571
(Q1/Q3)	(−4/1)	(−2/2)	(−2/3)	(−2/2)				
mean	−1.33	0.35	0.38	−0.4				
(SD)	(5.53)	(4.53)	(4.09)	(4.57)				

* Wilcoxon-test

### Changes in the relation between MacNew HRQOL and mental distress

MacNew HRQOL and HADS Total scores were negatively correlated. Over the whole observational period this correlation could be well described by a linear regression with MacNew Global score as dependent and HADS Total score as independent variable (p < 0.001). The coefficient of determination (R^2^) of the regression equation increased drastically from 0.45 at the baseline to a level between 0.67 and 0.77 in the follow-up period (Table 4). A separate analysis showed that the strengthening of the correlation between MacNew Global and HADS Total was based on all three MacNew subdomains: The coefficient of determination increased between baseline and follow-up in the emotional domain (from 0.57 to 0.76-0.80) as well as in the physical domain (from 0.24 to 0.50-0.56) and the social domain (from 0.32 to 0.57-0.69).

**Table 4:** Linear regressions with MacNew Global score as dependent and HADS total score as independent variable

	**Coefficient of determination - R**^**2**^	**Regression const. (SE)**	**Regression coefficient (SE)**	**p**
baseline	0.447	6.110 (0.107)	−0.104 (0.009)	<0.001
1 month	0.692	6.585 (0.067)	−0.117 (0.006)	<0.001
6 months	0.773	6.751 (0.058)	−0.122 (0.005)	<0.001
12 months	0.667	6.687 (0.075)	−0.117 (0.006)	<0.001
24 months	0.691	6.670 (0.068)	−0.116 (0.006)	<0.001

*SE* standard error

Looking at clinically relevant improving and stable patients in the time frame of 6 months after PCI separately by applying the MID cut-off point for MacNew HRQOL of 0.5, our data show that the improving patients move between baseline and follow-up towards a state of equilibrium between MacNew Global score and HADS Total score, which is in stable patients basically already present at the baseline (Additional file 2).

## Discussion

### Changes of MacNew HRQOL and HADS total score after PCI

Our data showed that MacNew HRQOL increases significantly after PCI. Focusing on intra-individual change over a 24 months period we found that major part of the improvement took place within the first month. Up to 6 months after PCI we noticed an additional increase in the physical and social MacNew subscale. From 6 months up to 24 months all MacNew scores remained stable. Therefore we conclude that the assessment of the direct impact of PCI on HRQOL has to cover a time frame of more than one but not more than six months. This conclusion is also supported by looking at the percentage of patients, in which improvement achieved or exceeded the minimal important difference (MID): 48.5% of the patients improved by 0.5 or more on MacNew Global score between baseline and 1 month; 14% of the patients, who have not improved in the first month after PCI, showed such improvement between baseline and 6 months. At the 6 months and the 12 months follow-up the percentage of newly improving patients stayed below 5%. This is the same level in which clinically relevant deterioration relative to the baseline occurred. Therefore it seems reasonable to assume that clinical meaningful change at 12 and 24 months follow-up should be considered as fluctuation unrelated to the coronary intervention at the baseline. Our data confirm the results of previous studies with MacNew [6,7,9,15] and other measures for HRQOL [4], which have demonstrated that HRQOL increases significantly after PCI. To our knowledge there is up to now no study focusing on the temporal pattern of intra-individual changes in HRQOL after PCI, but recent studies reporting HRQOL mean scores over a series of several points of follow up in large samples are consistent with our results: Weintraub and colleagues [20] reported improving HRQOL scores up to 3 months after PCI and subsequently stable scores up to 36 months using the Seattle Angina Questionnaire (SAQ); Cohen and colleagues [21] reported significantly improved SAQ scores at 6 and 12 months after PCI.

With regard to HADS total score we found a small but statistically significant decrease between baseline and 1 month follow-up. Afterwards the level of mental distress remained stable. Similar results were reported by Höfer and colleagues covering a 3 months follow-up period [7], by Damen and colleagues [22], who found only very small changes in HADS scores and a stable level of anxiety and depression between 1 month and 12 months after PCI and by Astin and colleagues [23], who registered a decrease in trait and state anxiety between pre-PCI and 6–8 weeks post-PCI and stable scores between 6–8 weeks up to 6–8 months using the Spielberger State Trait Anxiety Inventory.

### Relation between MacNew HRQOL and HADS mental distress

The most important finding of our study concerns the increase in the explanatory power of the linear regression with MacNew Global as dependent and HADS Total as independent variable from baseline to the follow-up period. This increase was based on all three MacNew domains (emotional, social and physical). Whereas at the baseline 45% of the variance of MacNew Global scores could be explained referring to HADS Total score this percentage rose to the remarkably high value of 77% after six months. Later on, 12 and 24 months after PCI, a decline to a level of 67% and 69%, respectively, occurred, which may probably be due to fluctuations of the scores unrelated to initial impact of coronary intervention at the baseline. Yohannes and colleagues [12] briefly mentioned a similar strengthening of the correlation between MacNew and HADS from the baseline (46%) to 12 months after treatment for myocardial infarction (79%) in their study on the long-term benefits of cardiac rehabilitation without discussing this finding in detail.

Our data suggest that the equation describing the relation between MacNew Global and HADS Total in the follow-up period is defining a state of equilibrium. In absence of actual symptoms of CAD both measures reflect basically the general health status and the general attitude underlying the self-assessment of health of individual patients. It seems reasonable to assume that the equilibrium between the two outcome measures in the follow-up period essentially resembles the state before the patients experienced actual symptoms of CAD. At the baseline the symptoms of actual CAD cause an imbalance, because they have a more pronounced impact on the disease specific MacNew HRQOL score than on the non-disease specific HADS score. The changes in both measures occurring as a result of PCI lead to the restoring of the equilibrium. Patients, who did not experience a clinically meaningful improvement of HRQOL after PCI, stayed for the most part already at the baseline at the state of equilibrium. They did not improve, because, as we assume, they never had deteriorated in the first place. This interpretation corresponds to previous findings in a study on illness perception schemata and their association with depression and quality of life by Le Grande and colleagues [24]. They found that a great proportion of cardiac patients is largely uninvolved with their disease. Further research focusing on illness perception especially with longitudinal study design is needed to gain a better understanding, why patients after coronary intervention strongly differ with regard to changes in HRQOL and mental distress.

Our study has several limitations. In order to describe individual changes in MacNew and HADS scores we included only patients, who answered all four follow-up questionnaires in time (i.e.: 53%). The large proportion of excluded cases is the downside of having as many points follow-up over a relatively long period of 24 month. However, the relatively small sample size of 163 patients is adequate to detect clinically important changes of HRQOL (i.e. ≥MID) and a separate analysis including all patients showed no substantial difference in mean values and with regard to the correlation between the two outcome measures at the respective point of follow-up. Another limitation is that we have not included data about the clinical outcome. Further studies with larger samples are needed to investigate the impact of adverse clinical events on HRQOL and mental distress after PCI. Due to the fact that the study was attached to a Xience stent register all patients were treated with Xience stents, which potentially reduces the generality of our results.

## Conclusion

Our study shows that the relationship between MacNew HRQOL and HADS Total in the follow up period in absence of actual symptoms of coronary artery disease represents a state of equilibrium, which is imbalanced at baseline. This finding is potentially consequential for the assessment of Quality of life changes. The state of equilibrium in the follow-up period provides a point of reference, which could be useful in evaluating treatment success in individual patients as well as in study populations.

## Abbreviations

CABG: Coronary artery bypass graft; CAD: Coronary artery disease; HADS: Hospital Anxiety and Depression Scale; HRQOL: Health Related Quality of Life; MID: Minimal important difference; PCI: Percutaneou*s* coronary intervention; SAQ: Seattle Angina Questionnaire.

## Competing interests

The authors have no competing financial or non-financial interests to declare.

## Authors’ contributions

JS and OF drafted the manuscript and were responsible for study design. Statistical analysis was performed by OF. SH, WB, TC and GG participated in conception and final revision of the manuscript. All authors read and approved the final manuscript.

## Supplementary Material

Additional file 1 Supplementary table: MacNew and HADS Total scores (t0: baseline; t1: 1 month; t2: 6 months; t3: 12 months, t4: 24 months). Click here for file

Additional file 2 **MacNew Global and HADS Total score at the baseline (A) and 6 months after PCI (B).** In the follow-up period clinically relevant improving patients are moving towards a state of equilibrium between MacNew Global and HADS Total score, which is basically already present in stable patients at the baseline. Click here for file
