# Supplementary material for: Health related quality of life and mental distress after PCI: restoring a state of equilibrium
Source: Health Qual Life Outcomes. 2013 Aug 27;11:144. doi: 10.1186/1477-7525-11-144 (PMC3765885; doi:10.1186/1477-7525-11-144)
Supplement: Additional file 1 — Supplementary table: MacNew and HADS Total scores (t0: baseline; t1: 1 month; t2: 6 months; t3: 12 months, t4: 24 months). [file 1477-7525-11-144-S1.docx]

Supplementary table

MacNew and HADS Total scores (t0: baseline; t1: 1 month; t2: 6 months; t3: 12 months, t4: 24 months)

|  |  | t0 | t1 | t2 | t3 | t4 |
| --- | --- | --- | --- | --- | --- | --- |
| MacNew Emotional | median  (Q1/Q3) | 5.14  (4.86/6.36) | 5.86  (4.86/6.36) | 5.86  (5.00/6.36) | 5.86  (4.93/6.43) | 5.79 (4.86/6.43) |
|  | mean  (SD) | 5.12 | 5.59  (1.03) | 5.60  (1.00) | 5.57  (1.07) | 5.60  (1.03) |
| MacNew Physical | median  (Q1/Q3) | 5.08  (4.25/6.00) | 5.85  (4.92/6.50) | 6.00  (5.16/6.54) | 6.00  (5.15/6.50) | 6.00  (4.98/6.55) |
|  | mean  (SD) | 5.04  (1.16) | 5.62  (1.11) | 5.75  (1.05) | 5.64  (1.12) | 5.68  (1.09) |
| MacNew Social | median  (Q1/Q3) | 5.38  (4.54/6.31) | 6.15  (5.60/6.62) | 6.31  (5.60/6.77) | 6.23  (5.38/6.77) | 6.31  (5.15/6.69) |
|  | mean  (SD) | 5.33  (1.17) | 5.80  (1.03) | 5.98  (1.00) | 5.88  (1.08) | 5.90  (1.03) |
| MacNew Global | median  (Q1/Q3) | 5.19  (4.44/5.89) | 5.85  (4.92/6.37) | 5.85  (5.15/6.38) | 5.89  (5.12/6.44) | 5.93  (4.96/6.42) |
|  | mean  (SD) | 5.11  (1.06) | 5.61  (1.00) | 5.69  (0.96) | 5.64  (1.04) | 5.67  (0.99) |
| HADS  Total | median  (Q1/Q3) | 9  (4/13) | 7  (3/12) | 8  (3/13) | 7  (3/14) | 6  (3/13) |
|  | mean  (SD) | 9.62  (6.79) | 8.29  (7.12) | 8.64  (6.90) | 9.02  (7.32) | 8.61  (7.14) |
