# Supplementary figures and images for: Health related quality of life and mental distress after PCI: restoring a state of equilibrium
Source: Health Qual Life Outcomes. 2013 Aug 27;11:144. doi: 10.1186/1477-7525-11-144 (PMC3765885; doi:10.1186/1477-7525-11-144)

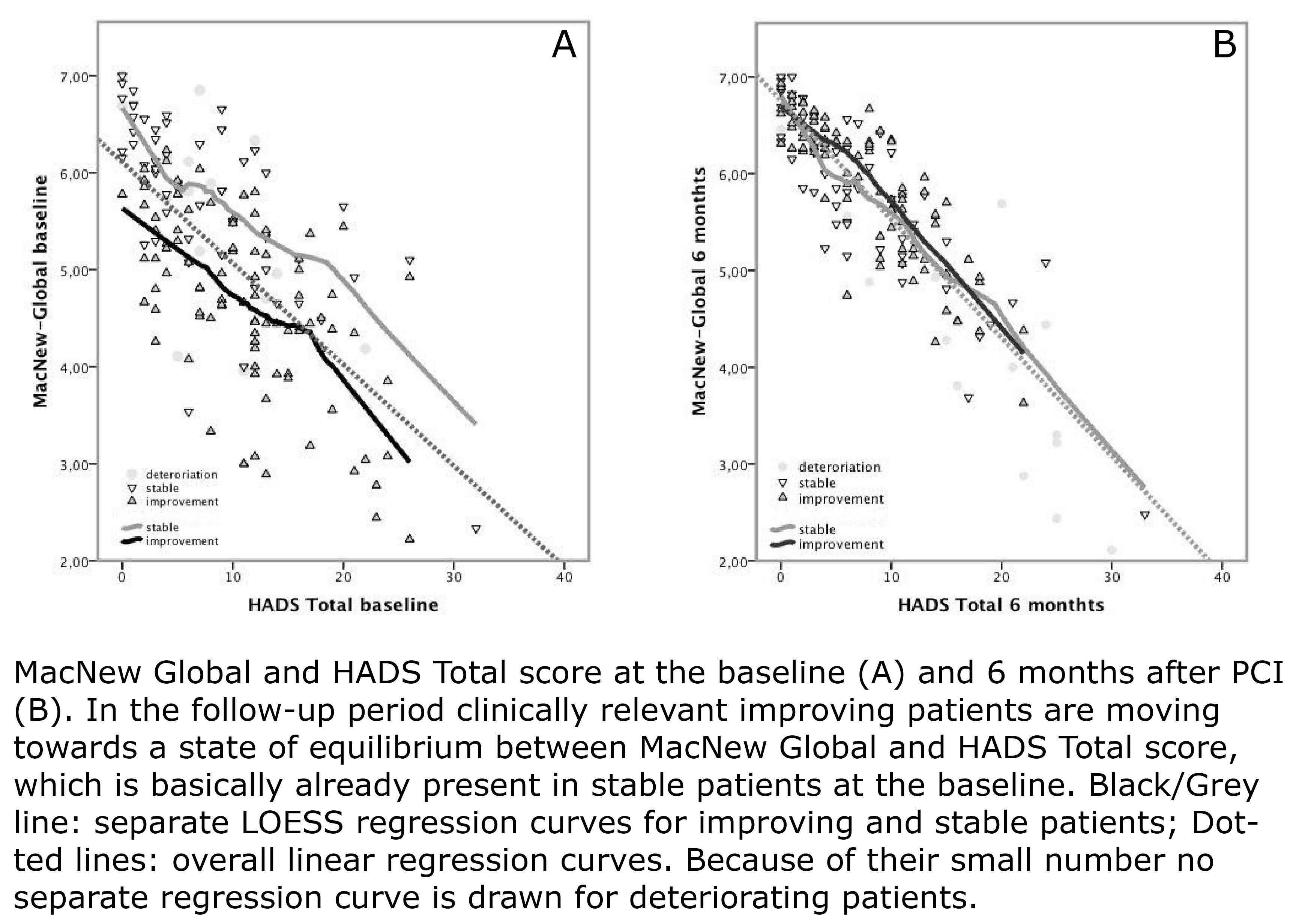

Supplement: Additional file 2 — MacNew Global and HADS Total score at the baseline (A) and 6 months after PCI (B). In the follow-up period clinically relevant improving patients are moving towards a state of equilibrium between MacNew Global and HADS Total score, which is basically already present in stable patients at the baseline. [file 1477-7525-11-144-S2.jpeg]
